# The effect of synbiotic coating of flaxseed mucilage‐defatted rice bran carbohydrate on quality of dried mango, viability of *Bifidobacterium animalis* subsp. *Lactis* BB12 on storage and simulating gastrointestinal condition

**DOI:** 10.1002/fsn3.4206

**Published:** 2024-05-13

**Authors:** Zohreh Didar, Mohammad Mehdi Soltan‐Dallal, Behzad Goharjoo

**Affiliations:** ^1^ Department of Food Science and Technology, Neyshabur Branch Islamic Azad University Neyshabur Iran; ^2^ Department of Pathobiology, School of Public Health Tehran University of Medical Sciences Tehran Iran; ^3^ Food Microbiology Research Centre Tehran University of Medical Sciences Tehran Iran; ^4^ Bachelor Student in TEFL Farhangian University Mashhad Iran

**Keywords:** *Bifidobacterium animalis* subsp. *Lactis* BB12, defatted rice bran carbohydrate, flaxseed mucilage, synbiotic

## Abstract

In the present study, a synbiotic coating of flaxseed mucilage, defatted rice bran carbohydrate, and *Bifidobacterium animalis* subsp. *Lactis* BB12 was fabricated for coating dried mango slices (M‐P‐C). The control samples contained only probiotic bacteria without coating (M‐P). Several quality parameters (moisture, weight loss, shrinkage percentage, pH, firmness, and color) were assessed on specific storage circumstances (25°C, relative humidity (RH) = 22%.). In addition, the survival of *Bifidobacterium animalis* subsp. *Lactis* BB12 was evaluated on storage and under simulated gastrointestinal (GI) conditions. According to the results, the log number of *Bifidobacterium animalis* subsp. *Lactis* BB12 reached 8.1 and 6.2 for coated and uncoated samples, respectively, during the 45 days storage at 25°C (>6 log CFU (log colony‐forming units)/g) and at finished stage of in vitro gastrointestinal circumstances, the log number of probiotic bacterial count reached 6.8 and 4 for coated and uncoated samples, respectively. The coating resulted in significantly less weight loss, moisture loss, and shrinkage of the mango slices than uncoated ones (*p* < .05). The growth of yeasts and molds was undetectable in both samples. The results of acceptance experiments for M‐P and M‐P‐C dried mango samples showedthat there were no significant differences between M‐P and M‐P‐C samples (*p*  >.05), indeed in the case of purchase intention and overall acceptability. After reading the text highlighting, there was no significant difference in all attributes of M‐P‐C samples pre and post of reading text highlighting. It could be concluded that the synbiotic coating of mango slices improved the quality characteristics of the dried mango as well as viability of the probiotic bacteria at storage time and under simulated gastrointestinal conditions.

## Graphical abstract

A synbiotic coating of flaxseed mucilage, defatted rice bran carbohydrate, and *Bifidobacterium animalis* subsp. *Lactis* BB12 was fabricated for coating dried mango slices. Quality parameters (moisture, weight loss, shrinkage percentage, pH, firmness, and color) were evaluated during storage at 25°C and relative humidity (RH) = 22%. In addition, the survival of *Bifidobacterium animalis* subsp. *Lactis* BB12 was evaluated during storage and under simulated gastrointestinal (GI) conditions.

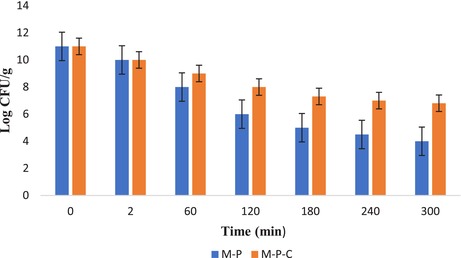

## 1 INTRODUCTION

Since the various drawbacks associated with polymeric packaging materials, films produced from edible components have gained focus by researchers (Gere et al., 2019). Apart from the protective effect of edible packaging materials, they could have a role as carrier of bioactive components like antimicrobial substances, probiotic bacteria, and various active components (Benbettaïeb et al., 2019), which could protect the food product as well as promote the consumer health (Pop et al., 2020).

The edible coatings are suitable support to mediate antimicrobial agents and antioxidants that potentially hinder the propagation of microorganisms and omit adverse oxidative degradability, respectively (Hani Tabaie Zavareh & Ardestani, 2020; Yaashikaa et al., 2023).

Recent novelty in the case of edible films and coatings is functional biopackages, like pathogen inhibitors and food preservatives, which help the food industry confer fresh, desirable, good‐quality food with useful health characteristics to consumers. The bioactive components from biological resources, especially those from byproducts, might have a remarkable role. Another novel approach includes applying specific microorganisms in edible films and coatings to inhibit the growth of pathogenic microorganisms. Probiotics are microorganisms with specific health benefits (Martins et al., 2024).

For the reason of achieving the profit from the consumption of probiotic bacteria, it is important that a magnitude of viable cells equal to 10^8–9^ per day is consumed. There are some challenges to reach this number of probiotics in food products such as high susceptibility of probiotics to environmental circumstances (Soukoulis et al., 2014).

The role of prebiotics in the gastrointestinal tract (GIT) is providing necessary nutrients to bacteria. Their action is crucial for maintaining the viability of probiotics in the digestive tract (Mohanty et al., 2018). The main prebiotics includes nondigestible oligosaccharides. Other components, such as stable starch and some peptides, proteins, and lipids, are also considered as prebiotic (Peng et al., 2020). Prebiotics must have some specific properties, such as: (1) hydrolysis by mammalian enzymes, stability against gastric acidity, and gastrointestinal (GI) absorption, (2) could be fermented by the gut microflora, and (3) favor the action and/or growth of health‐associated gut bacteria (Ceylan & Atasoy, 2023). The term “synbiotic” is composed of probiotics and prebiotics that profitability influence the host by modifying the stability for live microbial nutrition supplements in the digestive tract. (Mohanty et al., 2018). The synchronous application of probiotic bacteria and prebiotic components has achieved remarkable result. So, a desirable synchronous application of probiotic bacteria and prebiotic compounds in a product resulted in an excellent impact rather than usage of each one (probiotic or prebiotic) alone (Markowiak & Śliżewska, 2017).

In spite of decades of study on probiotics and prebiotics, it is a challenge to receive consistent desirable clinical consequences with these products. Surely, part of this is owing to wide categories of probiotic species and strains, the versatility in prebiotic structures, difference in doses of probiotics and prebiotics, and the particular target consequences that are defined in these researches. Furthermore, another noticeable subject is ecological limitations that affect the ability of probiotic microbes or prebiotic components to effect changes in the GI tract. The probiotics are oftentimes outnumbered a million‐to‐one by the commensal microbiota. For plenty of probiotic products, the microbes are not of human source and lack the required traits to compete and persist in the GI tract (Gomez Quintero et al., 2022). In particular, the capability to capture and use nondigestible carbohydrates and other specific resources is a remarkable parameter that determines if a given microbe could occupy a niche, even transiently in the GI environment. The usefulness of prebiotics alone is also subject to resemble personalized restrictions. For instance, for prebiotics to serve as optional component, related microbes should be able to use those substrates and convert them into useful products or other outputs to the host, which must be available in an individual's microbiome. It is also feasible that components that lack adequate structural or chemical specificity, instead, elevate nontargeted enrichment of members of the gut microbiome, which causes a lack of useful impacts on the host (Gomez Quintero et al., 2022). In conclusion, these ecologically noticeable subjects as well as immunological, physiological, and other host parameters contribute to the nonresponder phenotype generally observed across various researches and study populations (Ojima et al., 2022). So, one of the benefits of the synbiotic concept is the feasibility to solve these ecological problems by getting the microbe along with a component that supports the growth of that microbe (Swanson et al., 2020).

Synbiotics profit the host by exerting useful impacts via metabolite production, immune system regulation, and neurotransmitter production regulation (Sánchez et al., 2017). Eor et al. (2021) focused on the investigation of anti‐inflammatory and anti‐oxidative potential of synbiotics in two independent cell lines (colon cancer cells (HT‐29) as a gut epithelial cell and brain neuroblastoma cells (SH‐SY5Y) as a brain neuronal cell, respectively). In their study, 10 *Lactobacillus* strains with prebiotics were treated on two cell lines that assumed them as gut and brain when the host uses synergistic synbiotics. Selected synergistic synbiotics candidate remarkably protected each cell against various stressors (Eor et al., 2021).

Another advantage of utilization of dietary compounds, probiotics and prebiotics, is enhancing the immune system via their impacts on gut microbiota composition (Wypych et al., 2017).

Ozcan and Eroglu (2022) assessed the effect of stevia and inulin interactions on the fermentation profile of *Lactobacillus acidophilus* in milk and in vitro systems. They concluded that the addition of stevia enhanced the growth and activity of probiotic bacteria and ensured that the number of probiotic bacteria in yoghurts was maintained at the desirable magnitude to see the therapeutic impact and adding inulin in addition to stevia was more successful in enhancing the growth and amount of bacteria than applying stevia alone (Ozcan & Eroglu, 2022).

Food products are suitable carriers of probiotics for human utilization, even better than supplements, due to the food products supporting probiotics during the flow through the gastrointestinal tract (GIT), which is associated with their buffering characteristics and the existence of nutrients that can be utilized by the microorganisms. In fact, propagation and viability of probiotics are also influenced by physical and chemical characteristics of food matrices. In this respect, several food products, such as dairy products, meat products, fruits and vegetables, and low water activity foods, were evaluated (Soares et al., 2023).

Rice bran is produced during the processing of bran and is a by‐product. Rice bran is rich in carbohydrates, proteins, lipids, vitamins, minerals, dietary fiber, and some bioactive components (Antunes et al., 2023). Researches affirmed that rice bran carbohydrates appear to show good modulatory impacts on the gut microbiota and can be proposed of having a prebiotic impact and so as a possible new prebiotic could be assessed (Sapwarobol et al., 2021). Antunes et al. (2023) reported that the majority of the extract of defatted rice bran consisted of glucose, stachyose, and arabinose. Defatted rice bran carbohydrates were stable against simulated gastrointestinal circumstances and were able to promote the growth of some probiotic bacteria. They also showed good prebiotic activity for all *Lactobacillus* strains. These observations illustrated that defatted rice bran carbohydrates could be presumed as a possible source of prebiotics (Antunes et al., 2023).

Flaxseed or linseed (*Linum usitatissimum*) possesses approximately 6% mucilage (Cui et al., 1994). Flaxseed mucilage (FSM) could be applied as a gelling substance or thickener, and it has limited foaming characteristics (Puligundla & Lim, 2022).

It is also affirmed that carbohydrates included in the flaxseed mucilage have a specific biological role. These compounds have a desirable impact on the growth of probiotic *Bifidobacterium* strains (Courtois, 2009), have prebiotic characteristics, and are remarked as suitable additives for probiotic encapsulating component (Bustamante et al., 2017). According to the finding of the Bustamante et al. (2017), encapsulation of *Bifidobacterium infantis* and *Lactobacillus plantarum*, in the blend of maltodextrin along with mucilage and soluble protein of chia seed and flaxseed, resulted in a great viable rate of probiotic bacteria in the simulated gastric juice and bile solution as well as after 45 days of storage under refrigeration condition (Bustamante et al., 2017). The inclusion of pure flaxseed mucilage to kefir samples remarkably increased the viable count of lactic acid bacteria at the end of storage time (Veeramani et al., 2018). Blending flaxseed mucilage and gum arabic has been reported to enhance the viable rate of *Lactobacillus acidophilus* and *Bifidobacterium lactis* in kefir (Alhssan et al., 2023).

Sungatullina et al. (2023) reported the positive impact of flaxseed mucilage (FSM) mixing (at a content of 0.1%, 0.2%, and 0.4%) to de Man, Rogosa, and Sharp (MRS) and milk whey nutrient medium on the viability rate and other characteristics of *Lactobacillus bulgaricus*, *Lactobacillus fermentum* AG8, and *Lactobacillus plantarum* AG9. Accordingly, flaxseed mucilage can offer as a bioactive additive that can enhance antioxidant capacity and improve the stability and viability of *Lactobacillus* cells in the gastrointestinal tract (GIT) (Sungatullina et al., 2023).

Mangoes (*Mangifera indica*) has a high nutritional value and appealing organoleptic characteristics but this is a seasonal fruit and highly susceptible to spoilage, which results in remarkable postharvest damages. So, it seems important to introduce suitable operation technologies, like dehydration, to reduce the loss of this product (Mongi, 2023).

Mangoes (*Mangifera indica*L.) are one of the most significant tropical fruits in 2020, with global exports developing by 2.9%, and 60,000 tons, from the previous year. The global shipments of mangoes continue to make up about 90% of all exports (FAO, 2021). Globally, mangoes grow on about 3.7 million hectares (Jantuma et al., 2023). It was reported that the highest postharvest losses at farm level were approximately equal to 7.04%. Losses at wholesale market containing transportation were about 4.70%. Losses at retailing market, at storage unit, and at consumer level were about 3.66%, 3.50%, and 3.50%, respectively. It was also reported that the postharvest losses of processing were 3.11%. Final postharvest losses in mango at various steps from harvesting to consumption were approximately equal to 25.51% (Yusuf Ali et al., 2019). The drying of the mango appears as an interesting approach to diminish the losses postharvest (Kanyinda, 2017)

The aim of this research is investigation of the influence of symbiotic coating fabricated from flaxseed mucilage and defatted rice bran carbohydrate and added with *Bifidobacterium animalis* subsp. *Lactis* BB12 on the quality characterization of dried mango during storage and simulated gastrointestinal condition.

## 2 MATERIALS AND METHODS

### 2.1 Materials

Flaxseeds and rice bran were bought from a market and authenticated by the Department of Systematic Plant Biology, Islamic Azad University, Neyshabur. Rice bran was defatted by hexane (Surin et al., 2020). All chemical materials were purchased from Merck Company.

### 2.2 Defatted rice bran preparation

Rice bran powders was pretreated by applying the solvent (hexane) at a ratio of rice bran to solvent of 1:3 (w/v) by refluxing twice. The residue was dried at 40°C for 24 h (Surin et al., 2018).

### 2.3 Extraction of flaxseed mucilage

Water extraction approach was chosen for the extraction of flaxseed mucilage as per the following condition: stirring: 180 rpm (revolutions per minute), flaxseed to deionized water ratio of 1:8 (w/v), at ambient temperature, 18 h. The next step was vacuum filtration. The final steps were freeze‐drying and milling the mucilage (Sungatullina et al., 2023).

### 2.4 Extraction of defatted rice bran carbohydrates

Extraction of carbohydrates from defatted rice bran was accomplished, according to the method of Surin et al. (2020), via ultrasonic‐assisted extraction. For this reason, an ultrasonic bath (Eurosonic 4D, Italy) was applied as per the following condition: The ratio of defatted rice bran to water was equal to 1:20 w/v, temperature at 70°C, and time equal to 20 min (Surin et al., 2020). After extraction, centrifugation and evaporation were applied. Thereafter, removal of the starch and protein from the supernatants was done. Afterwards, three volumes of ethanol (95%) were blended with precipitate and centrifugation was carried out for the collection of the precipitate and washing was done with absolute ethanol. The next step was, to dialyze the precipitate first with tap water and next with distilled water. Finally, drying of the obtained polysaccharide was performed in a vacuum oven and packed in an aluminum foil laminated polyethylene pouch and stored at 4°C for next stages of experiments (Surin et al., 2020).

### 2.5 Probiotic bacteria preparation

*Bifidobacterium animalis* subsp. *Lactis* BB12 was kept at −20°C in MRS broth (Merck) and sterile glycerol stock (33% glycerol). Before each analysis, the strain was subcultured in MRS broth (37°C, 48 h under anaerobic circumstances). Thereafter, centrifugation was carried out (1200 × g, 10 min) and the obtained pellets were washed with sterile distilled water (Campaniello et al., 2020).

### 2.6 Mango preparation

First, the peeled sliced mango fruits (2–4 mm thick) were dried (90°C,60 min) in a force convection oven. Then, the samples were turned upside down and dehydration was continued for 60 min under similar circumstances (La Cava et al., 2022).

### 2.7 Preparation of the coated samples

Preparation of the coating solution was carried out on the basis of the method outlined by La Cava et al. (2022) with some modifications. Coating solution that included flaxseed mucilage (0.4%) and defatted rice bran carbohydrate (0.1%) was prepared. Bacterial pellets were blended within the solution to obtain a total count equal to 10^10^–10^11^ CFU/mL, and the pH was regulated to 6.0 with 30 min stirring. One hundred microliters of the obtained mixture was spread on to the dried mangoes that were dried in a forced convection oven (50°C/30 min) (La Cava et al., 2022). This sample was named M‐P‐C.

### 2.8 Preparation of uncoated samples

The uncoated samples were prepared as per the following steps: the prepared mango pieces were used as support materials and dipped into 250 mL of cell suspensions (10^10^–10^11^ CFU/mL) for 15 minutes (Campaniello et al., 2020). This sample was defined as the M‐P sample.

### 2.9 Evaluation of the final weight loss and characterization of the samples

For determination of the magnitude of weight loss, mango slices were weighed by a digital weighing scale (Laboratory Scale Model EK‐6100i, Japan). The final weight loss was calculated as per the following equation.

$$\text{Final weight loss}\;\left(\%\right)=\frac{\Delta \text{weight}}{\text{weight}\;\mathrm{on}\;\text{the first}\;\mathrm{day}}\times 100$$

The color of the mango slices was measured by a colorimeter (Laboratory color meter, YS6060 model). The color of samples was reported as L* (lightness), a* (redness–greenness), and b* (blueness–yellowness) magnitudes.

For the measurement of pH, 70 g of sample was mixed with 100 mL distilled water and after 12 h, filtration was performed and the pH was determined by a pH meter (Clean, pH 500).

### 2.10 Microbial assessment of the mango slices

First, the mango slices were homogenized with phosphate‐buffered saline (PBS) (with a ratio equal to 1:9 wt) (2 min) applying a stomacher. The homogenized samples were subjected to serial dilution (up to 10‐fold) and counted on MRS agar (Merck) by the pour plate method, and incubation was performed at 37°C, 48 h. For counting yeasts and molds, the spread plating approach on potato dextrose agar (Merck) containing 10,000 ppm (parts per million) of chloramphenicol was applied (Sigma‐Aldrich). Incubation at 30°C for 72 h was done. Counting was performed and the total counts were reported as logarithmic colony‐forming units per gram (log CFU/g) of mango (Wong et al., 2021).

### 2.11 In vitro digestion of mango slices

Both mango sample groups were exposed to simulated in vitro digestion. The fabrication of simulated salivary fluid (SSF), simulated gastric fluid (SGF), and simulated intestinal fluid (SIF) was carried out, according to Wong et al. (2021). First, 3 g of mango was minced under sterile circumstances. The minced mango was blended with 3 mL SSF and then placed in a shaking water bath with the following circumstances: 200 rpm, 37°C, 2 min. Survival of *Bifidobacterium animalis* subsp. *Lactis* BB12 was thereafter evaluated by counting on MRS agar plates (37°C, 48 h).

The obtained solution achieved after simulated oral step was blended with simulated gastric fluid at 1:1 ratio and the pH of the gastric chyme blend was adapted to 2.0 using hydrochloric acid (HCl) (6.0 M), and next the solution was placed in a shaking water bath with the following circumstances: 200 rpm, 37°C, 120 min. Bacterial enumeration was carried out on MRS agar at 60 and 120 min, respectively. The obtained solution from this stage was blended at 1:1 ratio with simulated intestinal fluid along with regulation of the pH to 7.0 with sodium hydroxide (NaOH) (6.0 M). Afterwards, the solution was placed on a shaking water bath (200 rpm, 37°C for further 180 min). Bacterial counts on MRS agar were carried out at specific intervals (180, 240, and 300 min) (Wong et al., 2021).

### 2.12 Storage stability

Fifteen grams of both sample groups (uncoated and coated) was located into glass desiccators and for reaching the RH equal to 22%, saturated solutions of potassium acetate (CH_3_COOK) were used. The samples were kept at 25°C for 45 days and analyzed for various parameters at 15 days intervals (La Cava et al., 2022).

### 2.13 Moisture content (%)

The moisture content was measured by weight loss during drying in a vacuum oven at 70°C, until reaching a constant weight. The moisture amount was reported as grams of water per 100 g of dried sample (d.s.) (La Cava et al., 2022).

### 2.14 Texture analysis

The texture of samples was assessed by texture analyzer (TA‐XT PlusTM, Stable Micro Systems). The measurement condition was as follows: spherical probe (5 mm diameter) and rate:1 mm/s (La Cava et al., 2022).

### 2.15 Scanning electronic microscopy (SEM)

Morphological observation of samples was assessed using SEM (Phenom ProX) under different magnifications (500–2500).

### 2.16 Determination of shrinkage magnitude

The shrinkage magnitude (%) was measured by determination of the variation in the bulk volume of the mango samples applying the liquid displacement approach using toluene and calculated by following formula (Ko et al., 2008).

$$\mathrm{Sh}=\frac{V0-V}{V0}\times 100$$

Where V_0_ and V are first and overall volumes of the sample (Noshad et al., 2014).

### 2.17 Ascorbic acid measurement

Determination of the ascorbic acid magnitude was accomplished by the approach of Parveez Zia & Alibas, 2021. Accordingly, after treatment, the samples were mixed with stabilizing buffer solutions. The next stage was addition of the filtrate with 2,6‐dichlorophenolindophenol solutions. Thereafter, the absorbance was read at 520 nm applying an Ultraviolet–Visible (UV–Vis) spectrophotometer (Jenway, Model 6300). The vitamin C magnitude was reported as milligrams per one hundred grams (mg/100 g) of dry matter (Parveez Zia & Alibas, 2021).

### 2.18 Antioxidant capacity and total phenolic content

For analysis of the antioxidant activity and the total phenol content (TPC), a specific amount of sample (0.5 g) was blended with methanol solution (volume:15 mL), concentration: 80% (v/v). Thereafter, the blended mixture was stirred (15 min) and next centrifugation was carried out (15 min, 8000 × g). After filtration of the supernatant section, the antioxidant activity was assessed with the DPPH (2,2‐diphenyl‐1‐picrylhydrazyl free radical) and outcomes were reported as milligrams of acid ascorbic equivalent antioxidant capacity (AAEAC)/100 g sample. TPC evaluation was accomplished applying Folin–Ciocalteu reagent. The standard for calibration was used as gallic acid (GA). The outcomes were showed as mg gallic acid equivalents (GAE)/100 g samples (La Cava et al., 2022).

### 2.19 Organoleptic characterization of samples and acceptance experiment

For the analysis of sensory characteristics of the two prepared sample groups, first, the panelists performed an acceptance experiment and purchase intention of M‐P and M‐P‐C dried mango samples. Thereafter, they performed the Text Highlighting, including an assessment of “like” and “dislike” information about dried mango, probiotics, synbiotics, and edible coating of flaxseed mucilage and the benefits/harmful related to them. M‐P‐C acceptance and purchase intention were assessed after the Text Highlighting to investigate the impact of information on M‐P‐C samples’ acceptance (Nogueira et al., 2023).

### 2.20 Text highlighting

This research used text highlighting in a direct application focusing on recognition and variation between M‐P and M‐P‐C mango samples to assess the consumer's perceptions about samples and evaluation of the information's effect on product acceptance. So, a text was written to give information about the concept of mango samples. This information was proposed to investigate consumer awareness and perceptions of the product. Aspects about the benefits/harms of consuming both sample groups were also included in the prepared text. The text (Table 1) included five paragraphs (each paragraph contemplated a main subtheme). It started by describing the nutritional value of dried mango. After, symbiotic products were described to provide major information to understand the variation between two dried mango groups. Then the beneficial impact of flaxseed mucilage was described. Paragraphs 4–5 described some potential awareness about the consumption of dried mango and symbiotic products, respectively.

**TABLE 1:** Text used in highlighting task and its main information content.

		Information content
Nutritional aspect	Paragraph1	Dried mango is a delicious and nutritious snack. It is a great source of vitamins and minerals, including vitamin C and fiber.
Beneficial aspects	Paragraph 2	The synbiotic coating on dried mangoes combines probiotics and prebiotics to create a powerful symbiotic relationship that supports gut health and overall well‐being. Synbiotic coating on dried mangoes offers to support healthy gut microbiome and enhanceg nutrient absorption
Nutritional viewpoint	Paragraph 3	Flaxseed mucilage is a soluble fiber found in flaxseeds that contain fiber, minerals, and bioactive components as well as show antioxidant activity (Kučka et al., 2024)
Potential undesirable effect of dried mango	Paragraph 4	There are some potential undesirable effects associated with dried mango: 1. High sugar content. 2. Caloric density: Dried mangoes are more calorie‐dense than fresh mangoes. 3. Dental health: The sticky texture of dried mangoes can cling on to teeth. 4. Mold growth: Improper storage of dried mangoes can lead to mold growth.
Potential disadvantage of symbiotic products	Paragraph 5	There are some potential disadvantages associated with symbiotic products that include: 1. Digestive issues. 2. Allergic reactions: Some individuals may be allergic to components of synbiotics (https://www.healthline.com/nutrition/probiotics‐side‐effects#TOC_TITLE_HDR_3, n.d.). 3. It might have Interactions with medications (Purdel et al., 2023)

A team included common consumers of dried mango who assessed the different organoleptic properties of samples. The panel consisted of 10 panelists who like dried mango and/or are common consumers of them. Samples were given a random code and presented individually in a random manner to each panelist. Properties that are evaluated by panelists consist of appearance, color, crispness, sweetness, and total acceptability by a hedonic approach with 9 points for each sample. The score of 1 implies extremely dislike and score equal to 9 implies extremely like (La Cava et al., 2022); (Campaniello et al., 2020).

The purchase intention was assessed by a 5‐point scale, ranging from “definitely would not buy” to “definitely would buy” (Nogueira et al., 2023).

First, panelists participate in the acceptance test and purchase intention of mango samples (M‐P and M‐P‐C). Serving order was regulated on the basis of the approach reported by Wakeling and Macfie (1995). Thereafter, panelists performed the Text Highlighting acceptance and purchase intention for M‐P‐C samples.

### 2.21 Statistical analyses

All examinations were performed in triplicate in different weeks. Analysis of variance (ANOVA), accomplished with least significant difference (LSD), was applied (*p* < .05). The analysis was performed using SPSS Software (version 29, SPSS Inc.) (Wong et al., 2021).

The Wilcoxon test analyzed acceptance and purchase intention data to compare M‐P and M‐P‐C dried mango sample outcomes before performing the Text Highlighting and to compare M‐P‐C acceptances pre‐ and post‐performing of the Text Highlighting. Results were shown by boxplot, using the median (Scudino et al., 2023). Analyses were performed using GraphPad (Prism, 8.0) software.

## 3 RESULTS AND DISCUSSION

Several properties of two sample groups were analyzed (moisture content, weight loss, pH, and shrinkage) and the results are shown in Table 2.

**TABLE 2:** Characterization of moisture content, weight loss (%), pH, and shrinkage (%) of samples during storage.

	Days	Moisture content (%)	Weight loss (%)	pH	Shrinkage (%)
M‐P	0	7 ± 0.1^a^	0^e^	3.7 ± 0.11^a^	42 ± 0.1^e^
	15	7 ± 0.1^a^	0.1^d^	3.7 ± 0.12^a^	44 ± 0.3^c^
	30	6.5 ± 0.1^b^	0.3 ± 0.01^b^	3.7 ± 0.10^a^	45 ± 0.2^b^
	45	6 ± 0.2^c^	0.4 ± 0.01^a^	3.7 ± 0.12^a^	46 ± 0.1^a^
	0	7 ± 0.15^a^	0^e^	3.7 ± 0.11^a^	42 ± 0.1^e^
M‐P‐C	15	7 ± 0.12^a^	0^e^	3.7 ± 0.11^a^	42.5 ± 0.1^de^
	30	6.9 ± 0.12^a^	0. 2 ± 0.01^c^	3.7 ± 0.13^a^	43 ± 0.1^d^
	45	6.7 ± 0.12^ab^	0. 2 ± 0.01^c^	3.7 ± 0.11^a^	44 ± 0.1^c^

*Note:* The values with different superscript letters in a column are significantly different (*p* < .05).

As seen in Table 2, the moisture content of two sample groups had no significant difference at initial day of storage (*p* < .05) but within storage time, especially longer duration of storage, some loss in moisture content was observed (Table 2). The magnitude of moisture loss during storage time in uncoated samples was remarkably higher than in coated samples (*p* < .05). A similar observation was obviously related to the percentage of weight loss (Table 2).

The magnitude of moisture content during storage time decreases and at the end of the 45 days, the moisture content reached 6 ± 0.2 and 6.7 ± 0.12 for M‐P and M‐P‐C samples, respectively. This result is in line with that of La Cava et al. (2022) who reported the magnitude of moisture content in mango snacks stored at RH 22% reduced during storage and attributed to a desorption process under such storage circumstances (La Cava et al., 2022). According to the National Standard of Iran for dried mango moisture content (The permitted moisture content for dried mango in this standard is lower than 8%) (Institute of Standards and Industrial Research of Iran. ISIRI 11080.1st. edition, 2008), both samples had a permitted level of moisture from this viewpoint. There was a decrease in weight of samples during storage especially at the end of storage time (Table 2), which might be related to the loss of moisture at the finished stage of storage (Table 2).

The measured pH of both samples was about 3.7 and there was no remarkable variation between both sample groups as well as during storage time (*p* < .05). Consistently, Mongi (2023) reported the pH of dried mango to be equal to 3.7 ± 0.20 in samples dried by a cabinet dryer and 3.6 ± 0.17 for samples dried in a tunnel dryer (Mongi, 2023).

The physiological weight loss was ascending to shrinkage (%), which might be owing to the evaporation or transpiration of moisture via the surface tissues and other biological fluctuations that occurred in the samples. The results of shrinkage (Table 2) showed the magnitude of the shrinkage percentage in both samples at initial day of experiment was equal to 42 ± 0.1 and there was no difference in the shrinkage percentage between two sample groups at day 0 (*p* < .05). This finding is similar to the observation of (Haneef et al., 2022). As the storage time increased, the magnitude of shrinkage percentage also increased especially in uncoated samples (Table 1). It might be concluded that coating acts as a barrier against moisture evaporation so the lower shrinkage was observed in coated samples (Haneef et al., 2022).

Analysis of texture property of samples (hardness) showed that there was no significant change in the hardness of both samples until 15 days of storage. Longer storage time (more than 15 days) resulted in enhancing the harness magnitude in both sample groups and the hardness in uncoated samples significantly was higher than that in coated ones (*p* < .05). This result is consistent with the moisture loss of samples during storage and it could be attributed to the loss of moisture caused hardness in the texture of samples.

Determination of the antioxidant activity of two sample groups was performed via DPPH method and the results are shown in Table 3. Accordingly, the initial magnitude of antioxidant activity of coated samples with flaxseed mucilage‐defatted rice bran carbohydrate was higher than that of the control sample (without coating). This might be due to the antioxidant capacity of flaxseed mucilage, which was affirmed by various researches (Safdar et al., 2020). During storage, there was a reduction in the antioxidant activity eighter in coated and uncoated samples and at the end of storage, the magnitude of antioxidant capacity reached 8.9 ± 0.3 and 6.5 ± 0.2 milligrams of acid ascorbic /100 g in coated and uncoated samples, respectively. Consistently, La Cava et al. (2022) also reported a decrease in antioxidant capacity in baked mango snacks during storage and attributed these results to the presence of oxygen in the air (La Cava et al., 2022).

**TABLE 3:** Characterization of textural property, antioxidant activity, total phenolic content, and ascorbic acid magnitude of samples during storage.

	Days	Hardness (g)	DPPH (milligram of acid ascorbic /100 g)	TPC (mg gallic acid equivalents (GAE)/100 g of sample)	Ascorbic acid (mg/100 g)
M‐P	0	250 ± 0.5^d^	9.5 ± 0.2^c^	110 ± 1^c^	6.8 ± 0.1^a^
	15	250 ± 0.7^d^	8.2 ± 0.1^e^	100 ± 1^d^	6.2 ± 0.1^d^
	30	253 ± 0.3^b^	7 ± 0.1^f^	95 ± 2^e^	5.9 ± 0.1^e^
	45	256 ± 0.2^a^	6.5 ± 0.2^g^	90 ± 2^f^	5.1 ± 0.1^f^
	0	250 ± 0.2^d^	10.5 ± 0.5^a^	115 ± 5^a^	6.8 ± 0.1^a^
M‐P‐C	15	250 ± 0.3^d^	9.7 ± 0.1^b^	112 ± 2^b^	6.7 ± 0.1^b^
	30	251 ± 0.2^c^	9.2 ± 0.1^d^	110 ± 1^c^	6.6 ± 0.1^ab^
	45	253 ± 0.2^b^	8.9 ± 0.3^de^	100 ± 2^d^	6.5 ± 0.1^c^

*Note:* The values with different superscript letters in a column are significantly different (*p* < .05).

Analysis of TPC in both samples revealed that the coated samples have more TPC content than the uncoated sample (Table 3). Some studies affirmed the presence of phenolic components in flaxseed mucilage and the major phenolic components in flaxseed mucilage include caffeic acid, p‐coumaric acid, epicatechin, ellagic acid, cinnamic acid, and vanillic acid (Vieira et al., 2019). The TPC of both mango samples showed a reduction in TPC content during storage but in coated samples reduction occurred lesser than uncoated samples (Table 3).

The ascorbic acid level of the uncoated sample was 6.8 ± 0.1 (mg/100 g) and reached 5.1 ± 0.1(mg/100 g) at the finished stage of storage (Table 3). Dried mango samples coated with flaxseed mucilage and defatted rice bran carbohydrate showed a lower decline in ascorbic acid content during storage than the control sample (*p* < .05). This observation is fitted with the finding of Muthmainnah et al., 2019 who showed coating chili with chitosan and gum arabic caused reduction in ascorbic acid loss (Muthmainnah et al. (2019)). Decrease in vitamin C content can be caused, owing to the occurrence of oxidative processes and the ongoing process of respiration (Muthmainnah et al. (2019)). Reduction in vitamin C magnitude is affected by oxidative processes. Several factors could stimulate oxidative processes, such as light, oxygen, heat, peroxide, and enzyme (Muthmainnah et al. (2019)).

### 3.1 The color analysis of samples

**FIGURE 1:**
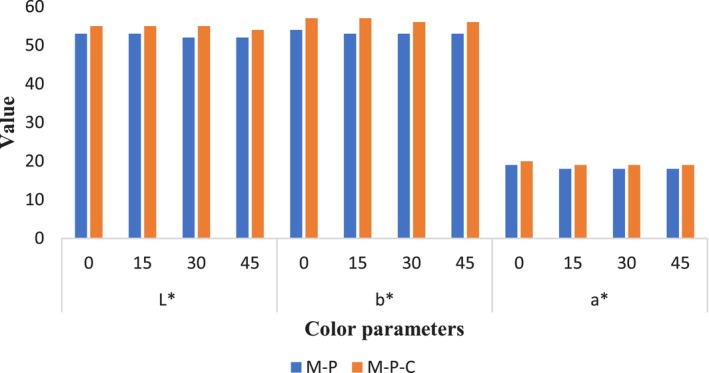
Color indices of samples: L* (lightness), b* (blue and yellow), and a* (red and green value).

The surface color of the samples was accomplished by applying a colorimeter (Laboratory color meter, YS6060 model). The color of samples was defined as L* (lightness), a* (redness–greenness), and b* (blueness–yellowness) magnitudes and the results are shown in Figure 1.

The color measurement implied that the lightness (L*) and yellowness (b*) were higher than a* values (Figure 1). This result is in a similar line with reports of other researchers (Izli et al., 2017; Khuwijitjaru et al., 2022). Accordingly, storage time has no significant change in all color indices (*p* > .05). Between two sample groups (M‐P and M‐P‐C), there was a slight difference in color indices that could be due to the coating formulation that changes the color indices in the surface of samples.

### 3.2 Microbial analysis of samples during storage

**FIGURE 2:**
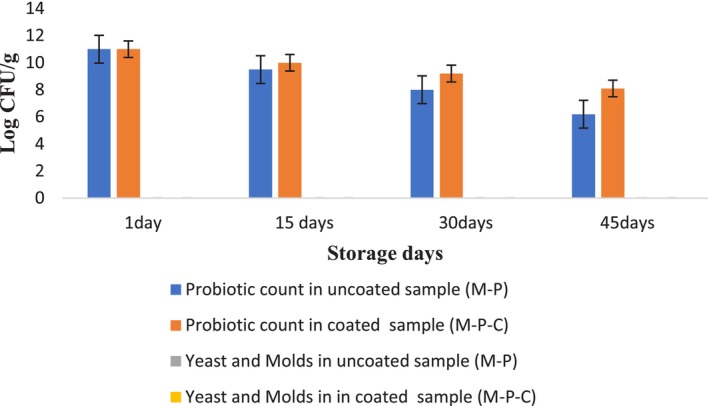
Probiotic, yeast, and mold counts in two sample groups during storage.

Figure 2 depicts the counts (log CFU/g) of probiotic bacteria, yeast, and molds in uncoated and the coated samples during storage.

Accordingly, the highest magnitude of viable *Bifidobacterium animalis* subsp. *Lactis BB12* all over the experiments (45 days) belonged to the mango slices coated with coating solution (Figure 2). As at the end of the storage period, the log total count of probiotic bacteria was equal to 8.1. Whereas, in the uncoated sample at the last stage of storage, the total log count of probiotic bacteria was 6.2. Both experiment samples have a probiotic count of more than 6 log CFU/g (the minimum desirable magnitude for probiotic presence declaration) (Hill et al., 2014).

The viability of probiotics was related to the kind of the chosen culture/probiotic kind, physiological condition of probiotic cells, food structure properties (e.g., acidity and a_w_), storage circumstances, existence of supportive vector, oxygen, and operation approach. Dianin et al. (2019) reported that the viability of *Lactobacillus casei* in the coating of whey protein isolate reaches the values from 7.5 to 5.7 log CFU/g after 28 days of storage at 25°C (Dianin et al., 2019). Marín et al. (2019) reported that the survival of *L. plantarum* coated with sodium caseinate after 7 days was 6.2 CFU/g (Marín et al., 2019).

Prebiotic substances have been approved to enhance the viable rate of probiotic bacteria on the operation of edible packaging (Pop et al., 2020). The inclusion of prebiotic substances in probiotic‐included packaging implies the desirable impacts on the resistance of immobilized probiotic bacteria (Soukoulis et al., 2014), Although, variation in coated fruit/vegetable composition, operation and stored circumstances, and species of probiotic bacteria and its population could affect the impacts of the prebiotic mixing on the viable rate of probiotic bacteria in films/coatings (Alvarez et al., 2021). La Cava et al. (2022) reported that snacks prepared with mango coated with microorganism blended in PBS and stored at 85% RH had a higher inactivation rate that revealed the supportive action of coating with pectin and calcium during storage (La Cava et al., 2022).

The high viability of probiotic cell in the present study might be attributed to the probable prebiotic characteristics of both flaxseed mucilage (Lai et al., 2021; Puligundla & Lim, 2022) and defatted rice bran carbohydrate (Antunes et al., 2023).

According to the results, there was no detectable growth of both yeast and molds during storage of both samples (Figure 2). This observation is made in accordance with similar studies that affirmed reduction in the growth of yeast and molds when various probiotic coatings were used in different fruit products. Shigematsu et al. (2018) showed the prevention of fungal growth at a storage time (19 days) of minimally processed carrot. Fruits with coating had less magnitude of mold and yeast counts at the end stage of storage time when using probiotic coating containing *Lactobacillus acidophilus* (Shigematsu et al., 2018).

Speranza et al. (2018) reported that the number of molds, yeasts, and psychrotrophic bacteria was lower than the limit of detection during the storage in apple and melon pieces containing probiotic coating of *Lactobacillus plantarum* (Speranza et al., 2018).

Hashemi and Jafarpour (2021) reported coated kiwi slices with probiotic bacteria (*L. plantarum* AF1, *L. plantarum* LU5, and *L. plantarum* LP3) had reduced mold and yeast counts (Hashemi & Jafarpour, 2021).

### 3.3 Bacterial count assessment on MRS agar plates (log CFU/g mango) on simulated in vitro digestion

**FIGURE 3:**
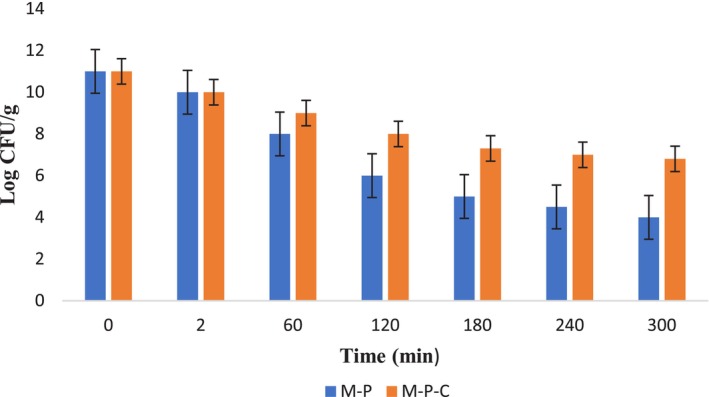
Viability of *Bifidobacterium animalis* subsp. *Lactis* BB12 during simulated digestion in both coated (M‐P‐C) and uncoated (M‐P) samples.

Figure 3 depicts the survival of *Bifidobacterium animalis* subsp. *Lactis* BB12 after each stage of the in vitro digestion, either for uncoated sample (M‐P) or for sample coated with synbiotic coat (M‐P‐C).

Accordingly, in simulated oral phase, the number of probiotic cells reached 10^10^ ± 0.1 in both samples and there was no remarkable difference between two tested samples related to the bacterial counts (*p* > .05). These results might be due to short exposure time and the neutral pH (equal to 7.0) in simulated oral condition (Wong et al., 2021).

After this stage, the simulated gastric condition was assessed and this step showed remarkable reduction in the cell count in both samples, especially the M‐P sample (Figure 3). The cell counts at 120 min in subjecting simulated gastric condition reached 10^6^ ± 0.2 and 10^8^ ± 0.1 in M‐P and M‐P‐C samples, respectively. This implies that the flaxseed mucilage‐defatted rice bran carbohydrate provided additional protection for probiotic cells against simulated gastric circumstances (Figure 3). The higher reduction in probiotic cell numbers in simulated gastric condition is due to the pH of SGF (equal to 2.0) and longer exposure time (120 min), (Wong et al., 2021). According to the results, it could be concluded that the coating provides a physical barrier that offers much better protection of probiotic cells rather than the uncoated sample that was obvious after 120 min (*p* < .05) (Figure 3). These results were compatible with the report of Wong et al. (2021) that affirmed physical protection of coating and the more stability of probiotic cells coated with edible coating than free cells (Wong et al., 2021).

At the simulated intestinal condition, the reduction of cell numbers continued specially in the uncoated sample (Figure 3). As in this step, the determined log count of probiotic cell was 4 and 6.8 in M‐P and M‐P‐C samples, respectively. Bile salts are capable of changing the structure of the proteins of the membranes of probiotic bacteria, thereby influencing the penetration of probiotics (Li, 2012). According to the results, incorporation of probiotic bacteria in flaxseed mucilage‐defatted rice bran carbohydrate could protect probiotic cell in simulated gastrointestinal condition and at the end of these circumstances, the overall cell count remained more than 10^6^ CFU/g (Figure 3). These results are in line with those of Wong et al. (2021) who reported more reduction of probiotic cells in simulated gastric condition rather than in simulated intestinal condition, as well as the free cells showing drasticreduction during simulated gastrointestinal condition than samples that included edible coating (Wong et al., 2021).

### 3.4 Scanning electron microscopy (SEM)

**FIGURE 4:**
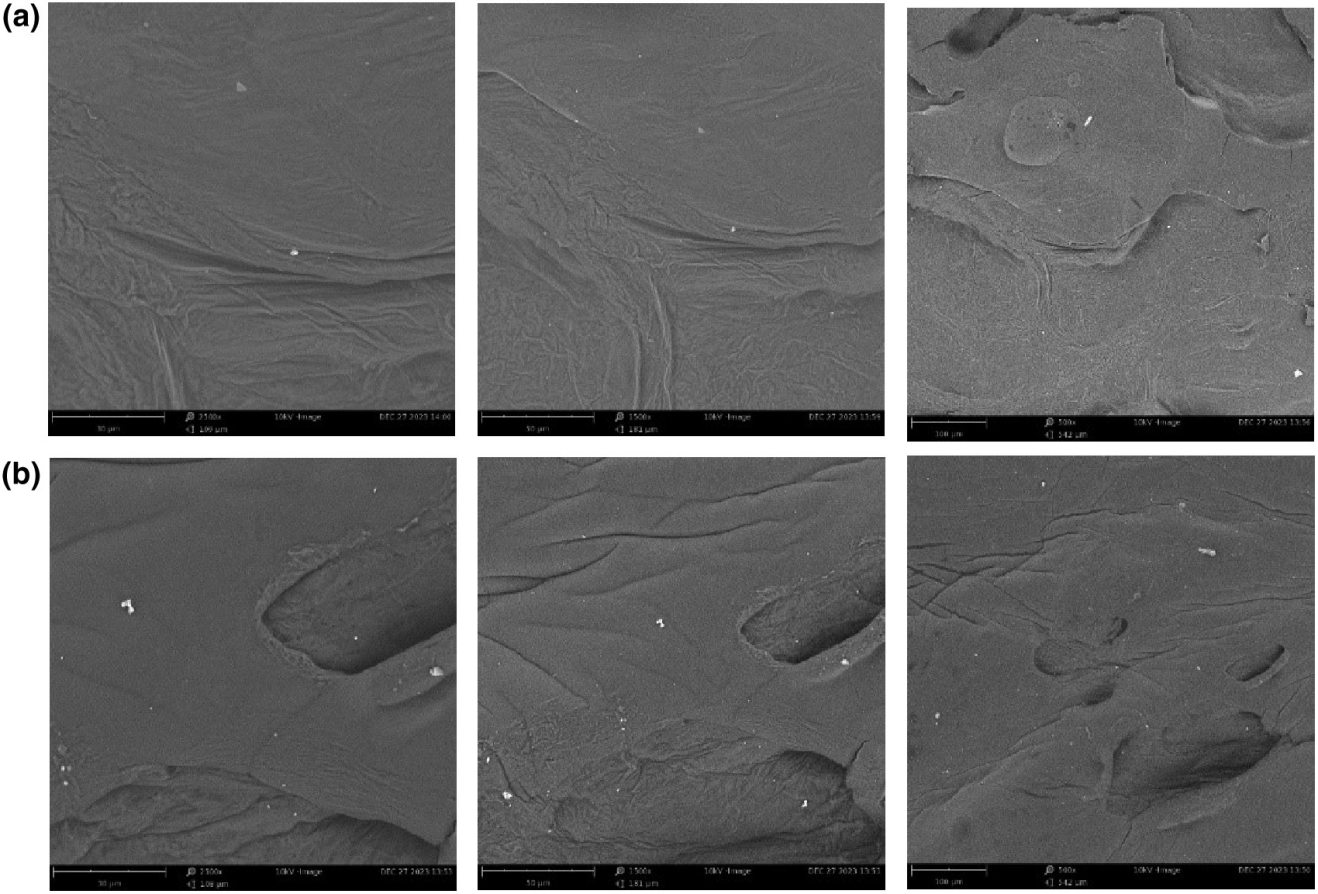
Scanning electron microscopy (SEM) images of uncoated (a) and coated (b) samples.

Surface morphological assessment was performed by utilization of the SEM instrument (Phenom ProX, Netherlands) at various magnifications and the images are shown in Figure.4. Accordingly, both sample groups had a homogeneous and compact structure. Their structures were quite compact, owing to the contraction with a slightly rough surface due to the drying process.

### 3.5 Sensory evaluation

**FIGURE 5:**
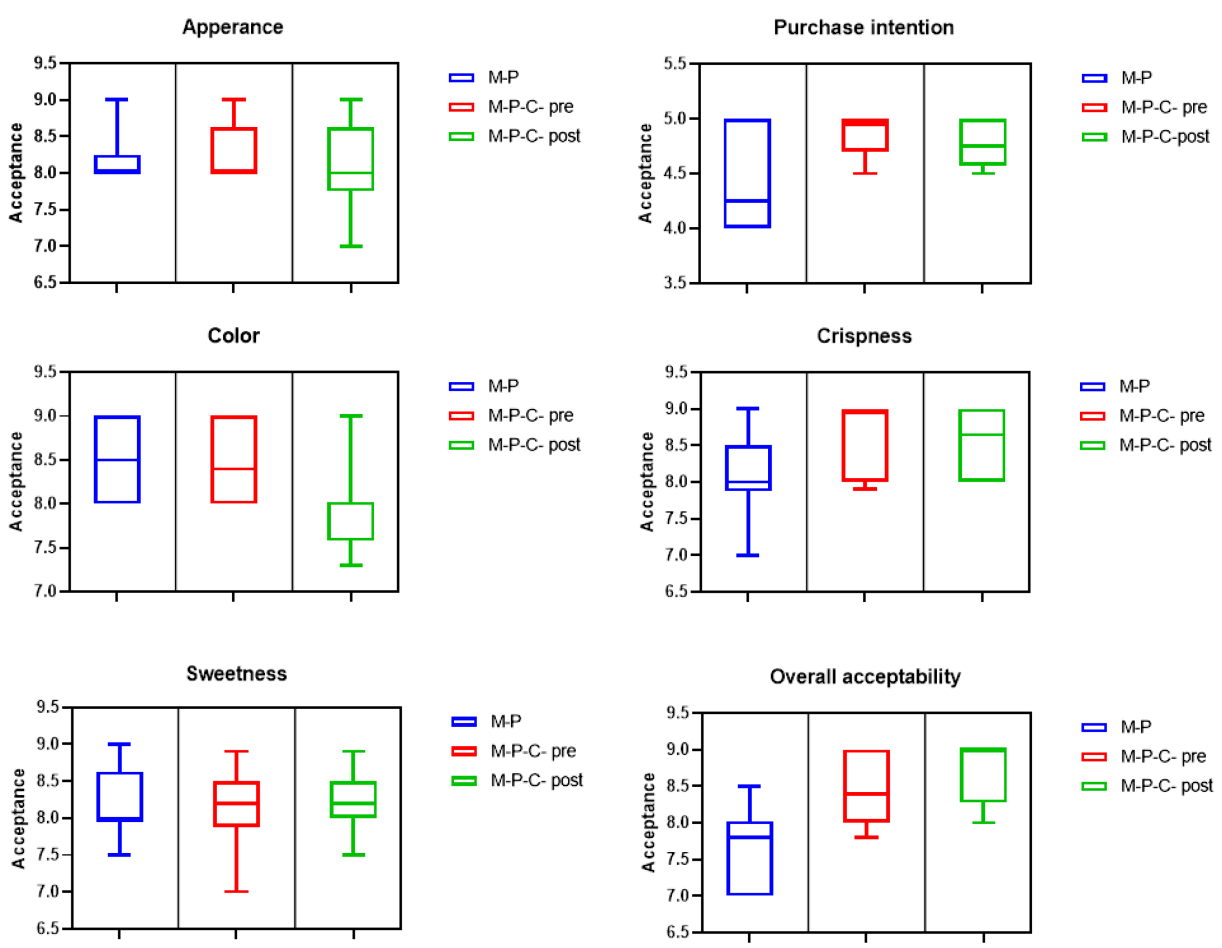
Sensory acceptance of M‐P and M‐P‐C samples without information (M‐P‐C‐pre); and after reading the text (M‐P‐C‐post).

A group (10 panelists) included regular consumers of dried mango who assessed the different organoleptic properties (appearance, color, crispness, sweetness, and overall acceptability) of samples by 9‐point hedonic approach. Figure 5 and Table 4 depict the acceptance outcomes achieved for M‐P and M‐P‐C dried mango samples; and M‐P‐C acceptance after reading the text (after performing the Text Highlighting).

**TABLE 4:** Comparison of the acceptance results obtained for M‐P and M‐P‐C samples pre and post of reading the text.

Sensory attribute	Comparison	*p* value
Appearance	M‐P× M‐P‐C	.619^ns^
Color	M‐P× M‐P‐C	.652^ns^
Crispness	M‐P× M‐P‐C	.063^ns^
Sweetness	M‐P× M‐P‐C	.536^ns^
Overall acceptability	M‐P× M‐P‐C	.014^a^
Purchase intention	M‐P× M‐P‐C	.008^a^
Appearance	M‐P‐C × M‐P‐C	.443^ns^
Color	M‐P‐C × M‐P‐C	.059^ns^
Crispness	M‐P‐C × M‐P‐C	.446^ns^
Sweetness	M‐P‐C × M‐P‐C	.343^ns^
Overall acceptability	M‐P‐C × M‐P‐C	.105^ns^
Purchase intention	M‐P‐C × M‐P‐C	.310^ns^

*Note*: ^a^Significant difference and ^ns^ No significant difference at 5% of probability by Wilcoxon test.

According to the results, there were no significant differences between M‐P and M‐P‐C samples (*p* > .05), indeed, in the case of purchase intention, there was an obvious significant difference between M‐P and M‐P‐C sample (*p* = .008) (with mean equal to 4.41 and 4.86, respectively). The overall acceptability showed significant difference between M‐P and M‐P‐C samples (*p* = .014) with mean equal to 8.16 and 8.43, respectively (Table 4).

After reading the text highlighting, there was no significant difference in all attributes of M‐P‐C samples and no difference between purchase intention pre‐ and post of reading text highlighting (Table 4) (*p*  >0.05).

## 4 CONCLUSIONS

This research focused on the formulation of mango‐based carrier for *Bifidobacterium animalis* subsp. *Lactis* BB12. A synbiotic coating containing flaxseed mucilage and defatted rice bran carbohydrate was used for one group of samples and the other group prepared without coating. Several quality characterizations of both groups were assessed at initial and during 45 days of storage. Survival of probiotic bacteria at storage time and at simulated gastrointestinal condition was also evaluated. According to the results, the coated samples conferred better survival of probiotic bacteria at storage duration as well as at simulated gastrointestinal conditions that might be due to the prebiotic property of flaxseed mucilage and defatted rice bran carbohydrate. The acceptance outcomes were achieved for M‐P and M‐P‐C dried mango samples; and M‐P‐C acceptance after reading the text (after performing the Text Highlighting) and results showed there were no significant differences between M‐P and M‐P‐C samples (*p* > .05), indeed, in the case of purchase intention and overall acceptability. After reading the text highlighting, there was no significant difference in all attributes of M‐P‐C samples and no difference between purchase intention pre and post of reading text highlighting.

## AUTHOR CONTRIBUTIONS

**Zohreh Didar:** Conceptualization (equal); data curation (equal); formal analysis (equal); funding acquisition (equal); investigation (equal); methodology (equal); project administration (equal); writing – original draft (equal); writing – review and editing (equal). **Mohammad Mehdi Soltan‐Dallal:** Resources (equal); software (equal); supervision (equal); validation (equal); visualization (equal); writing – review and editing (equal). **Behzad Goharjoo:** Writing – review and editing (supporting).

## FUNDING INFORMATION

There is no funding to declare.

## ETHICS STATEMENT

Not applicable.

## CONSENT TO PARTICIPATE

Authors read and approved the final manuscript.

## CONSENT FOR PUBLICATION

Authors have read and agreed to the published version of the manuscript.

## CODE AVAILABILITY

Not applicable.

## Data Availability

Data are available upon request.
